# Identification of cancer-specific cell surface targets for CAR-T cell therapy

**DOI:** 10.1186/s41232-024-00329-2

**Published:** 2024-03-29

**Authors:** Naoki Hosen

**Affiliations:** Department of Hematology and Oncology, Osaka University Graduate School of Medicine, 2-2, Yamada-Oka, Suita, 565-0871 Osaka Japan

**Keywords:** CAR-T cell, Lineage-specific antigens, Post-translational modification

## Abstract

One should identify appropriate cell surface targets to develop new CAR-T cells. Currently, lineage-specific antigens such as CD19 or B cell maturation antigen (BCMA) are being used as targets for CAR-T cells. However, in most cancers, lineage-specific antigens cannot be used as targets because targeting normal counterparts expressing them causes fatal toxicity. Cancer-specific transcripts have been extensively searched for using transcriptome analysis, but only a few candidates were reported. We have been working on identifying tumor-specific antigen structures, for example constitutively activated conformer of integrin b7 in multiple myeloma. Recently, several researchers have been working on a logic gate system that can react only when two antigens are expressed on the cell surface.

## Background

Chimeric antigen receptor (CAR)-T cell therapy targeting CD19 received approval in the USA in 2017 and in Japan in 2019. Due to its remarkable therapeutic efficacy, its clinical application is advancing more rapidly than initially anticipated. The structure of CAR-T cells comprises an extracellular region that specifically binds to the target antigen, a hinge region connecting the extracellular and intracellular segments, a transmembrane region, and an intracellular region responsible for activating the T cells. Here we provide a comprehensive review of the targets of CAR-T cell therapy.

## Main text

### What are the targets of CAR-T cells?

CARs must detect antigens with high sensitivity and specificity, and CAR-T cells should selectively harm cells expressing these antigens. The targeted antigen for CAR-T cells must meet two criteria: (1) expression on cancer cells, including cancer stem cells, and (2) absence on normal cells crucial for survival.

#### Lineage-specific antigens

Currently, the approved and clinically practiced target antigens for CAR-T cells are CD19 and B cell maturation antigens (BCMA), both classified as lineage-specific antigens. Given that CD19 is expressed on B cells, and BCMA on both B cells and plasma cells, patients undergoing CAR-T cell therapies targeting these antigens experience damage to normal B cells and plasma cells. Nevertheless, this is not fatal, as the deficiency in humoral immunity, a crucial function of B cells and plasma cells, can be compensated for through supplementation with immunoglobulin by infusion.

Many cancers exhibit antigens shared with their normal cell counterparts. However, in most cases, those antigens cannot be used as targets because there are only a few normal cells whose complete loss would not be fatal to the overall life homeostasis. B cells and plasma cells are rare exceptions. For example, myeloid leukemia poses challenges for targeting myeloid lineage-specific antigens such as CD33 or CLL1 because the total loss of their normal counterparts, myeloid cells, would be lethal. How about solid tumors in this context? Certain solid organs can be resected while maintaining viability, for example prostate gland. Consequently, PSMA, specific to prostate cells, serves as an antigen for prostate cancer [1].

#### Antigens with high specificity for cancer

As previously mentioned, lineage-specific antigens cannot serve as viable targets for most cancers. Therefore, it becomes crucial to identify antigens specifically expressed in cancer for CAR-T cell development. However, targets for CARs require a high degree of specificity, unlike antibody drug targets, and this is exemplified by the challenges in developing CAR-T cells against HER2, a widely utilized target for antibody drugs [2].

Despite these challenges, extensive efforts by numerous researchers have yielded reports of antigens demonstrating relatively high cancer specificity suitable for CAR-T cell targeting. One such example is GD2, which has shown effectiveness against neuroblastoma [3] and some glioblastomas [4] in clinical trials. Another promising target is Claudin18.2, identified for its efficacy against gastric cancer, with clinical trials of CAR-T cells targeting it demonstrating some effectiveness [5].

#### Cancer-specific genetic mutations and splicing abnormalities

Each patient possesses distinct mutated genes. For a mutated site to be a target antigen for CAR-T cells, it must be expressed on the cell surface. Consequently, the pool of widely applicable target mutant antigens is limited. Epidermal growth factor receptor splice variant III (EGFRvIII), a splicing variant with a partial extracellular region deletion, is found in many glioblastoma patients [6]. CAR-T cells designed to recognize EGFRvIII have demonstrated efficacy against glioblastoma. However, targeting EGFRvIII did not eliminate all tumor clones as it was not expressed uniformly on all tumor cells [7]. Thus, the difficulty lies in identifying widely available antigens derived from cancer cell mutations due to variations among cancer patients and high diversity within a single patient’s cancer cells. While extensive analyses of genetic mutations have been undertaken, recent revelations about splicing abnormalities in many cancers suggest that targeting splicing variants common among patients could streamline CAR-T cell development. Ongoing global research is likely to explore this avenue.

In the case of mutations in intracellular proteins, CAR cannot directly react with the mutated protein. However, peptides derived from mutated proteins can be presented on HLA. Thus, a complex of a neoantigen peptide and HLA may be a target for CAR-T cells. This strategy can be also used for non-mutated intracellular tumor antigens. Such TCR-like CAR is being developed and tested [8].

#### Cancer-specific post-translational changes of proteins

We have been endeavoring to identify cell surface antigens specific to multiple myeloma (MM). Since the global search for genes and proteins expressed exclusively in MM cells has been thoroughly conducted, the identification of novel MM-specific transcripts or proteins seems to be exceedingly challenging. However, cancer-specific antigen structures resulting from post-translational events, such as glycosylation, complex formation, or conformational changes, might have been overlooked in prior screenings. Notably, a cancer-specific glyco-epitope on the Muc1 protein (Tn-Muc1) has been demonstrated as an excellent target for CAR T cells against several types of cancers [9].

Antigen epitopes of this kind could be discovered by meticulously exploring cancer-specific monoclonal antibodies (mAbs) and characterizing the antigens they recognize. Therefore, we initiated the development of large numbers of mAbs binding to MM cells and sought mAbs binding exclusively to MM cells, not normal hematopoietic cells. Consequently, we identified an antibody named MMG49 as an MM-specific antibody from over 10,000 clones of mAbs binding to MM cells. Subsequently, we discovered that MMG49 binds to integrin β7. Intriguingly, MMG49 did not bind to normal lymphocytes despite integrin β7 being expressed in them. Further investigation revealed that MMG49 specifically binds to the active conformation of integrin β7, not the inactive conformation. The MMG49 epitope is located in the N-terminal region of the β7 chain, predicted to be inaccessible in the resting integrin conformer but exposed in the active conformation. Elevated expression and constitutive activation of integrin β7 conferred high reactivity of MMG49 on MM cells, whereas MMG49 binding was scarcely detectable in other cell types, including normal integrin β7^+^ lymphocytes. MMG49 is unlikely to bind to non-hematopoietic tissues since integrin β7 mRNA is not expressed in tissues other than blood cells. Additionally, the MMG49 antigen was highly expressed in CD19-positive clonotypic B cells, potential MM precursor cells, indicating it is a promising therapeutic target for eradicating entire MM clones. These results strongly suggest that the MMG49 antigen is an ideal target for CAR T cells against MM. T cells transduced with MMG49-derived CAR exhibited remarkable anti-MM effects without harming normal hematopoietic cells [10].

In the same screening, we also identified R8H283, a mAb binding to MM cells but not normal hematopoietic or non-hematopoietic cells. R8H283 specifically recognized CD98hc, binding to CD98hc in heterodimers with CD98 light chains, which function as amino acid transporters. MM cells abundantly expressed CD98 heterodimers to uptake amino acids for constitutive immunoglobulin production. Although CD98 heterodimers were also expressed in normal leukocytes, R8H283 did not react with them. Normal leukocytes expressed CD98hc glycoforms distinct from those in MM cells, possibly explaining the lack of R8H283 reactivity in normal leukocytes. R8H283 exerted significant anti-MM effects without damaging normal hematopoietic cells. These findings suggest that R8H283 is a new source for mAb-based therapies against MM [11].

**Fig. 1:**
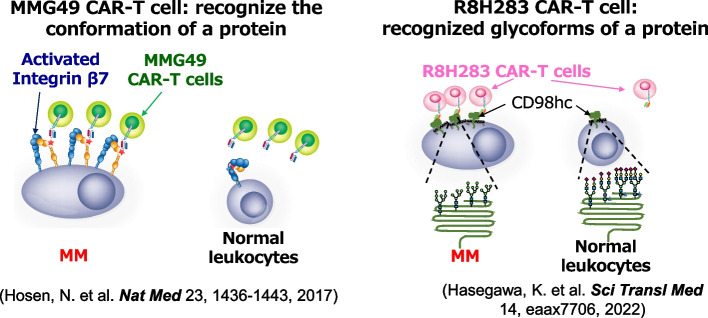
Cancer-specific conformational epitopes can be identified in non-tumor-specific proteins by extensive screening of primary human tumor samples

Our efforts have identified two myeloma-specific antibodies and their recognized antigen structures (Fig. 1). MMG49 targets the active conformation of integrin β7, permanently activated in myeloma cells. The R8H283 antibody recognizes CD98hc, a relatively widely expressed protein, with specificity likely attributable to differences in CD98hc glycosylation. A clinical trial of CAR-T cells derived from MMG49 is ongoing.

### Structure of the extracellular region of CAR

After determining the target, the choice of antibody to bind to it becomes crucial. CAR-T cells approved in Japan against CD19 use extracellular regions composed of FMC63, a mouse-derived monoclonal antibody [12]. Among BCMA-approved CAR-T cells, ide-cel employs a mouse antibody-derived scFv for antigen recognition, while cilta-cel uses a llama antibody-derived heavy chain sequence. Llama antibodies, devoid of light chains, recognize antigens with a single chain. Cilta-cel utilizes VHs from two different antibodies recognizing BCMA.

The specific antigen recognition domain is not necessarily an antibody-derived variable sequence. CAR-T cells using the ligand molecule of the target antigen as the recognition site have been developed, such as those recognizing GM-CSF receptors on leukemia cells using GM-CSF sequences, currently undergoing clinical trials [13].

Concerning the extracellular region beyond the target-binding segment, a linker connecting the light chain to the heavy chain and a hinge linking the light chain to the cell membrane exist. Reports indicate that the length of the linker and hinge influences T cell activation [14]. Given the target’s variability, optimization should be performed for each antigen recognition sequence site.

### Cancer-specific recognition using two antigens

While distinguishing cancer cells from normal cells using a single antigen poses challenges, numerous groups are exploring the potential of combining multiple antigens for enhanced specificity.

One approach involves using antigens A and B, each carrying distinct signals, wherein T cells are activated only when both signals are transmitted. A recent and intriguing development is the creation of CARs binding LAT to an scFv recognizing CD19 and SLP-76 to an scFv recognizing HER2, both integrated into a single T cell. Activation occurred only when both antigens were expressed, not when just one was present [15].

Another strategy is incorporating both an activating receptor and an inhibitory receptor into a single CAR-T cell. This is achieved by introducing a CAR recognizing and activating CEA, coupled with a CAR recognizing HLA A02 to inhibit T cell signaling. Cancer cells, in their attempts to evade patient immunity, may lose HLA at times. Consequently, CAR-T cells interacting with CEA-expressing cancer cells receive activation signals without inhibitory signals due to the absence of HLA A02. In contrast, normal cells expressing CEA receive inhibitory signals due to their expression of HLA A02 and remain unharmed [16].

Additionally, CAR-T cells employing an artificial receptor, such as the synthetic Notch receptor (synNotch), are capable of expressing new genes upon recognition of specific molecules [17]. For example, a synNotch receptor that recognizes EGFRvIII can be used to locally induce expression of CAR recognizing Ephrin A2 and IL13Ra2 to specifically target glioblastoma.

## Conclusions

CAR-T cell therapy has demonstrated remarkable success in treating certain hematological tumors. Its potential expansion to solid tumors and other diseases in the future underscores the critical importance of selecting appropriate targets and refining the extracellular domain of CAR-T cells. Similar to anticancer therapy, which often involves a combination of multiple agents, the evolution of CAR-T cell therapy against multiple targets is anticipated to enhance therapeutic efficacy and safety.

## Data Availability

Not applicable.
